# Real-World Evaluation of the Eye+Dot Online Triage Support Tool in Community Optometry Practices: Mixed Methods Evaluation Study

**DOI:** 10.2196/77278

**Published:** 2026-03-16

**Authors:** Jen O Lim, Sarah Farrell, Samuel J Newlands, Louise Allen

**Affiliations:** 1 School of Clinical Medicine, University of Cambridge, Cambridge, United Kingdom; 2 Department of Ophthalmology, Cambridge University Hospitals NHS Foundation Trust, Hills Road, Cambridge, CB2 0QQ, United Kingdom, 44 01223 216700

**Keywords:** triage, triage methods, mobile apps, computer-assisted decision making, decision tree, primary health care, emergency service, eye diseases, optometry, mobile health, digital health

## Abstract

**Background:**

Many patients attending hospital emergency services with recent-onset eye symptoms could have been managed in the community. This may reflect a lack of specialist experience or triage capacity among primary care providers. Online triage tools collect patient symptomatology and relevant ophthalmic and medical history virtually, compile a report, and suggest an outcome decision which can support the streaming and direction of patients to a suitable service within an appropriate timeframe.

**Objective:**

Our objectives were to implement and analyze the real-world use of a novel online triage tool in a community optometry setting.

**Methods:**

This was a real-world mixed methods evaluation of a web app “eye+dot” (Cambridge Medical Innovation Ltd), which uses a conditional logic multiple-choice questionnaire to produce a symptom report and automated triage recommendation. A preimplementation survey was used to gather views regarding current triage practices and interest in trialing an online triage tool among community optometrists. The app was then implemented in participating practices, with optometrists receiving the symptom report but not the automated triage disposition. The sensitivity and specificity of optometrists and the app at identifying urgent cases (those requiring specialist examination within 24 h) were compared to a gold standard, generated by retrospective review of the symptom reports by hospital specialists. Metadata including patient age, test duration, and usability feedback from patients and optometry staff were analyzed.

**Results:**

Seven optometry practices trialed the app over 5 months. Records of 209 patients with a mean age of 53.5 (SD 17.5; range 13‐90) years were analyzed. Three patients with potentially sight- or life-threatening symptoms (as defined by Royal College of Ophthalmologists guidelines) received appropriate urgent hospital dispositions by both optometrists and eye+dot. The app’s automated disposition had similar sensitivity to optometry triage (75.5% vs 77.3% respectively; *P*=.99) but significantly better specificity (82.7% vs 54.4%; *P*<.001) for identification of the 53 cases categorized as requiring either hospital or community specialist examination within 24 hours by gold-standard review. A total of 147 patients gave acceptability feedback, with 121 (82%) of them giving a rating in the top 2 points of a 5-point visual Likert scale. Optometrists in 5 of the 7 practices found the app helpful and were interested in continuing its use.

**Conclusions:**

This real-world study demonstrates the triage tool’s accuracy for identifying high-risk symptomatology and its acceptability among patients and optometry services. The significantly lower optometric triage specificity may reflect risk-averse decision-making following direct communication with patients and appointment availability in primary care. In addition, the relatively low mean age of patients suggests possible barriers to uptake among older patients. This and other symptom-based triage tools can provide useful support to eye care providers in the community where access to specialist triage may be limited.

## Introduction

### Background

Patients with new eye or vision symptoms often contact community optometry practices in person or by telephone for advice. Informal triage and advice may be given if there is capacity to do so, but a lack of specific remuneration for triage activities and limited optometrist availability can mean that such patients are redirected to emergency departments (ED) or emergency eye clinics (EECs) with minimal specialist optometrist input. Community optometrist–delivered Minor Eye Condition Services (MECS) and Community Urgent Eyecare Services (CUES) have been commissioned in many areas of the United Kingdom to provide urgent, optometry-led community eye care and reduce pressure on emergency services, and studies indicate that between 37% and 92% of self or general practitioner-referred patients in EECs may be suitable for these services [1-5]. Efficient and early redirection of suitable patients to MECS or CUES could reduce the 870,000 annual ED attendances across the country for eye and vision symptoms and move care into the community, a key goal in the National Health Service (NHS) Long Term Plan [6].

Paper triage support charts are currently used by many optometry practices and EDs. More recently, patient-facing online symptom-based tools with automated triage algorithms have been developed [7-11]. Several have been validated with encouraging results in EDs, but to our knowledge, no digital specialist triage support tool for ophthalmic symptoms has been used to stream and direct patients in the community setting. However, evaluation studies of nonophthalmic digital triage tools in the primary care setting have provided promising results but reveal possible difficulties around triage accuracy, with automated tools often tending to unnecessarily overtriage cases as emergencies, and equity concerns around access and ease of using such online tools for older patients or patients with disabilities [12]. In addition, integration with existing workflows can be challenging, and low patient compliance with suggested triage advice can prevent gains in practice efficiency from being realized [13,14].

### Study Aims

The purpose of this study was to evaluate the potential use of an eye and vision symptom-based online triage support tool, examine its real-world implementation, and analyze its accuracy at identifying high-risk ophthalmic symptomatology in the community.

## Methods

### Study Design

This was a prospective, mixed methods real-world study focusing on how the technology works for patients in everyday community optometry practice. Real-world studies enable testing the technology with real patient scenarios and the inclusion of patients and practitioners who might otherwise not take part in research, making the results more useful for the appropriate context but less strictly controlled than formal validation studies.

### Ethical Considerations

Approval of the study’s methodology was obtained from the quality and safety committee of the Cambridge University Hospitals NHS Foundation Trust and the Cambridgeshire and Peterborough Integrated Care Service (approval reference: PRN10395). As an anonymized service evaluation that did not involve deviation from the current standard of care, this study was exempt from requiring national ethics committee approval [15]. Patients gave verbal agreement to participate and digitally agreed to the General Data Protection Regulation (GDPR) statement prior to undertaking the online eye+dot questionnaire, and the study was undertaken according to the tenets of the Declaration of Helsinki. Patients could choose to opt out without affecting their care.

### The Technology

The eye+dot web app (Cambridge Medical Innovation Ltd) is a triage support tool that uses a conditional logic multiple-choice questionnaire of up to 25 questions to collect relevant ophthalmic and clinical history, detect high-risk symptoms, compile a downloadable symptom report, and suggest a triage disposition in terms of appropriate service and urgency. The tool has been developed and iteratively improved over 4 sequential proof-of-concept and validation studies in 3 hospital trusts of the Cambridgeshire and Peterborough Integrated Care Service. The automated triage algorithm used in eye+dot was adapted from the symptom triage guidelines published by the Royal College of Ophthalmologists (RCOphth) [16]. The initial process involved the development of multiple-choice questions and the conditional logic structure. These, and the triage disposition algorithm, were tested and iteratively improved, initially using simulated clinical scenarios, and subsequently evaluation and validation in over 500 real clinical scenarios. Wording, accessibility, and user interface improvements have been made based on patient and clinician feedback. Recent integration with the Electronic Patient Record (Epic) enables clinicians to order and retrieve results directly through the patient record. The app is currently in clinical use in an NHS hospital trust. As the eye+dot app replicates a chart-based symptom scoring chart, it does not meet the criteria for classification as a Medical Device under the UK Medical Devices Regulations based on Medicines and Healthcare Products Regulatory Agency guidance.

**Figure 1.**
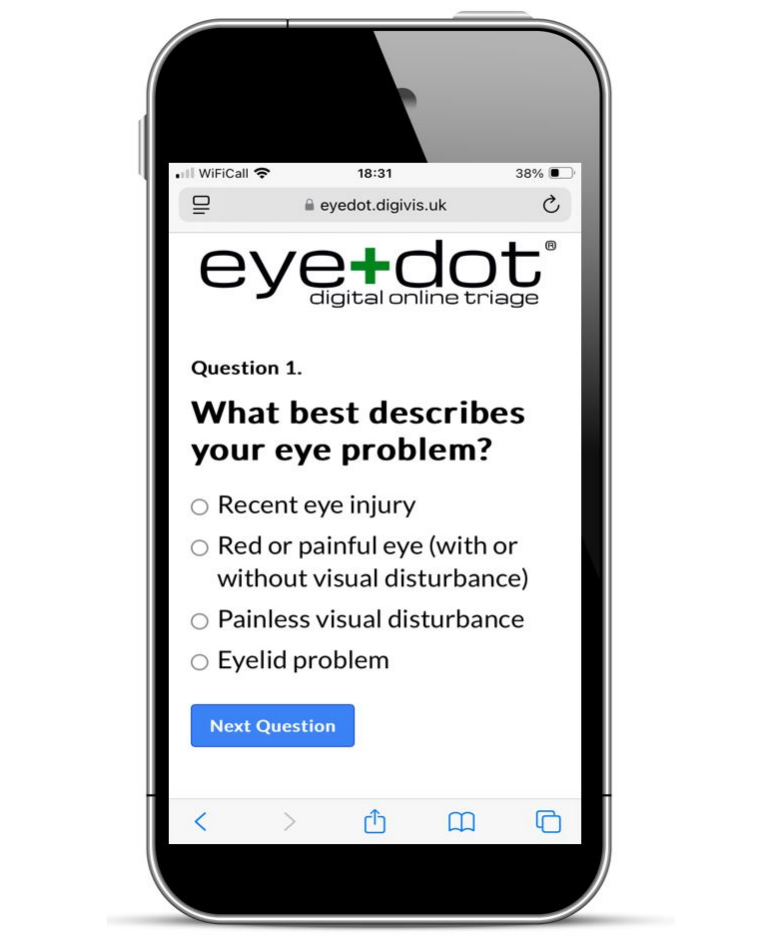
Initial filter question of the triage tool as it appears on a smartphone.

The care provider sends the patient a weblink to the online questionnaire by SMS text messaging or email, documenting the unique order number in the patient record. The patient accepts an initial standard disclaimer, GDPR-compliant data usage notice, and inputs their age before starting the questionnaire. The first filter question as seen by the patient is shown in Figure 1. Web browser translation is available but was not used for this study. Symptoms are automatically rated in terms of acuity adapted from previously published triage support tables (Multimedia Appendix 1) [16]. The symptom acuity rating creates an automated disposition, with the suggestion of an appropriate service (ED or EEC, MECS or CUES, and other community optometry services or pharmacist) and urgency (same d, within 24 h, within 48 h, and within a wk or routine). The symptom report and automated disposition are automatically emailed to the provider’s nominated inbox and can also be accessed via the secure online portal (Multimedia Appendix 2). This report can also be used to facilitate effective referrals, which other studies have shown are often affected by incomplete content [17,18]. The report is not routinely made available to the patient unless there is a history of potentially severe chemical, blunt, or high-velocity trauma. In such cases, the patient receives an immediate response with a weblink to the NHS first aid website and advice to attend emergency services urgently.

For the purposes of this study, the ordering provider received the symptom report but was masked to the automated disposition to prevent biasing of their clinical decision.

### Preimplementation Survey

Optometrists and managers in the 23 practices registered with the Cambridgeshire Local Optical Committee (a statutory body representing and supporting optometrists in our region) and accredited in the provision of specialist eyecare with our NHS hospital trust were invited to complete an online survey, designed using Microsoft Forms (Microsoft Corp), regarding current triage practices, and to express interest in participating in a 5-month pilot of a novel online triage tool (Multimedia Appendix 3). These practices serve a population of approximately 920,000. Survey questions were adapted from the validated Normalization Measure Development instrument based on the normalization process theory [19,20] and included questions about patient pathways and volumes, triage tools currently in use, and overall triage outcomes.

### Implementation

Practices interested in trialing the triage tool participated in a training webinar (led by LA and SF) to explain the use of the triage tool and the implementation process. Two similar webinars were held at different times of the week to encourage attendance, and a recording was made available online for those practice staff unable to attend. At these webinars, optometrists and administrative staff received instruction in how to order an eye+dot link for patients and complete a study data spreadsheet with the date, name of participating patients, eye+dot reference number, and the outcome of optometry triage. Spreadsheets were pseudonymized prior to being returned to the study team for analysis. Participating practices were asked to use the tool’s symptom report in addition to their current triage process for a 5-month period. Patients were directed to services based on their optometry practice’s triage decision rather than the eye+dot automated triage disposition.

Criteria for inclusion were that the patient should be aged 13 years or older with normal cognition, a good understanding of English, and recent-onset eye or vision symptoms (within 7 d). Patients may have either walked into the practice or phoned for advice. Patient selection was otherwise highly pragmatic, with practice staff inviting patients to undertake the online questionnaire in addition to the practice’s standard triage process. Only patients who agreed to using eye+dot were sent the link to the online tool’s questionnaire. Participation was voluntary and patients were informed that the standard of care would not be affected by either agreeing or declining to participate. A further written informed consent process was not necessary for this service evaluation study and was not carried out, although all patients using eye+dot were asked to consent to its GDPR-compliant data use policy before commencing the symptom questionnaire. Patients without digital access were able to undertake the questionnaire over the telephone, with the receptionist documenting the patient’s responses on their behalf. The generated symptom report was automatically returned to the nominated practice email inbox to be made available to the triaging optometrist. The unique weblink order number and the optometry triage outcome were recorded by a staff member in a spreadsheet held securely by the practice. To compensate practices for the administration involved, an honorarium of £15 (€17.5 or US $19) was agreed for each completed and deidentified patient record, payable at the end of the study period.

### Postimplementation Report Review

Following the 5-month period of implementation, records from optometry practices were deidentified prior to their release to the study team for analysis. Each patient’s online symptom report, automated triage disposition, and optometric triage outcome were linked with their unique eye+dot order number. Only patients meeting the eligibility criteria with recorded optometric triage outcome data and a complete online eye+dot report were included for analysis.

Two hospital-based specialists (consultant ophthalmologist LA and hospital optometrist SF), masked to the optometrist and eye+dot triage tool dispositions, reviewed the symptom reports and identified patients with high acuity symptoms who required either urgent hospital care or hospital or community specialist assessment within 24 hours. In cases of disagreement, a third clinician (specialist clinical fellow SN) made the casting decision. This was used as the gold standard triage disposition.

Postimplementation feedback from participating optometrists was communicated by email requesting free-text responses under the following domains: advantages and disadvantages of using eye+dot, barriers and enablers to its uptake, improvements that could be made, and likelihood of adoption in the future.

### Statistical Analysis

Preimplementation and postimplementation survey responses were analyzed using descriptive statistics. Differences in sensitivity and specificity of eye+dot automated triage and optometric triage at identifying high-acuity cases as defined above were evaluated using the McNemar test with continuity correction on GraphPad Prism version 10.4.1 (GraphPad Software). In addition, interrater reliability at identifying urgent or nonurgent cases between eye+dot and the other modes of triage, as well as between the 2 main reviewers who determined the gold-standard triage outcome, was evaluated by calculation of Cohen κ. A priori interpretation of κ values was as follows: values ≤0 indicating no agreement, 0.01 to 0.20 (none to slight), 0.21 to 0.40 (fair), 0.41 to 0.60 (moderate), 0.61 to 0.80 (substantial), and 0.81 to 1.00 (as almost perfect agreement). Additionally, adherence to RCOphth guidelines for hospital assessment within 24 hours for presentations suggestive of a sight or life-threatening condition (Multimedia Appendix 4) was assessed. Metadata including duration and completion of the online triage questionnaire, patient age, and feedback on the app using a voluntary visual Likert scale with responses converted to numerical values (Multimedia Appendix 5) were analyzed by descriptive statistics.

## Results

### Preimplementation Survey

The preimplementation survey (Multimedia Appendix 3) received 53 responses, 50 (94%) of which were from optometrists, and the remaining 3 from practice managers. From the 53 respondents, 36 (68%) reported that they or their practice currently used a paper-based triage tool, and 45 (85%) reported that they were the first point of contact for most patients with recent onset eye symptoms. The most frequent triage outcome prior to implementation of the online triage tool was reported as a General Ophthalmic Service or private appointment by 27 (51%) respondents, a MECS or CUES appointment by 23 (43%), and advice with or without purchase of eye drops by 2 (4%). One optometrist reported that the most common outcome was advice to attend ED or EEC.

From the 53 respondents, 45 (85%) agreed or strongly agreed that they would be willing to learn how to use an online triage tool, 39 (74%) agreed or strongly agreed that an online tool could improve patient care and make onward referral easier, and 30 (57%) felt that patients would be willing to undertake an online questionnaire for triage purposes.

### Implementation

The online triage tool was implemented in 7 out of 23 invited optometry practices comprising 4 Specsavers branches and 3 independent practices, all of which also provided MECS services. In total, 14 members of staff attended one of the live webinar sessions. Four other practices expressed interest but were unable to participate due to a lack of administrative or time resources.

**Figure 2.**
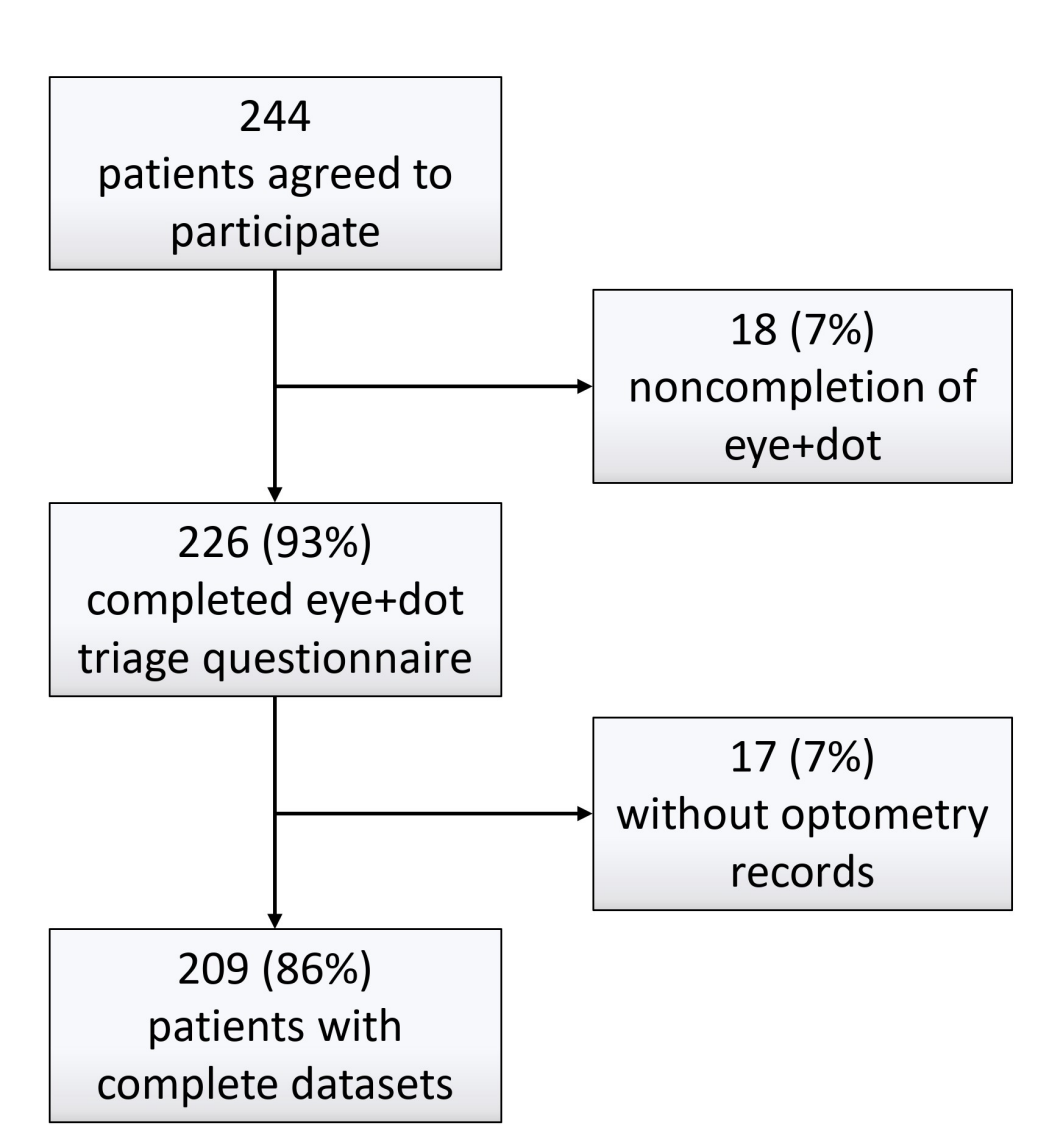
Implementation phase flowchart.

During the 5-month implementation period, 244 patients agreed to be sent the online triage link by their practice. Of the 244 patients, 18 (7%) started but failed to complete the online questionnaire, and a further 17 (7%) were excluded due to incomplete optometry triage outcome records. The remaining 209 records were analyzed (Figure 2). The mean age of patients was 53.5 (SD 17.5; range 13‐90) years. Of the 209 records, 136 (65%) included information on the mode of questionnaire completion. For 19 patients, this was undertaken over the phone with the help of a receptionist rather than online. Based on the responses to the first filter question of the online triage questionnaire, the most frequent primary presenting complaint was red or painful eyes, followed by painless visual disturbance, eyelid problems, and eye injuries (Table 1).

**Table 1.** Characteristics of patients with complete online and optometry triage records (N=209).

Feature	Patients, n (%)
Age range (y)	
<20	7 (3.3)
20‐29	22 (10.5)
30‐39	16 (7.7)
40‐49	27 (12.9)
50‐59	51 (24.4)
60‐69	48 (23)
70‐79	27 (12.9)
>79	11 (5.3)
Main presenting complaint	
Red and/or painful eye or eyes	95 (45.5)
Painless visual disturbance	82 (39.2)
Eyelid problems	22 (10.5)
Eye injury	12 (5.7)

### Triage Outcomes

Optometric triage outcomes (Multimedia Appendix 6): 106 out of 209 patients (51%) were seen within 24 hours. Of these, 4 (2% of 209) were advised to contact or attend ED or EEC and 31 (15% of 209) attended same-day optometry appointments. 89 (43% of 209) patients were offered less urgent optometry appointments, 13 (6% of 209) given advice or dispensed eyedrops without an appointment and 1 (1% of 209) advised to contact their general practitioner.

Eye+dot triage disposition (Multimedia Appendix 6): 67 out of 209 patients (32%) were allocated a disposition of hospital or community specialist assessment within 24 hours, and 9 (4% of 209) of these were recommended same day ED or EEC appointments. A total of 132 (63% of 209) patients received less urgent triage dispositions to hospital or community optometry services, while pharmacist advice was recommended for a further 10 patients.

### Accuracy at Identifying High Acuity Cases

From the 209 patients, 53 (25%) were considered to have potentially urgent eye problems requiring hospital or community specialist eye assessment within 24 hours according to the gold-standard triage dispositions established by expert review. This included 3 patients with presentations meeting the RCOphth definition of an emergency eye condition (Multimedia Appendix 4). There was no significant difference in the proportion of patients with gold-standard categorizations requiring specialist assessment within 24 hours presenting to Specsavers branches and independent optometry practices (25% vs 24% respectively; odds ratio 1.07, 95% CI 0.57‐2.02; *P*=.84).

All 3 patients with presentations meeting the RCOphth definition of an emergency eye condition [16] were correctly identified as requiring urgent hospital attendance by both optometric and automated triage mechanisms.

Of the 53 patients assigned a gold standard recommendation of hospital or community specialist assessment within 24 hours, 40 (76%) were assigned that outcome by eye+dot triage, with the remainder (n=13, 25%) assigned to be seen within 48 hours. Of the same 53 patients, 41 (77%) were assigned a hospital or optometrist assessment within 24 hours by optometry triage, with a further 4 (8%) to be seen within 48 hours, and 8 (15%) cases considered routine (Table 2). The online triage tool had similar sensitivity as optometry triage (75% vs 77%, respectively; *P*=.99) but significantly better specificity (83% vs 54%, *P*<.001) at identifying cases requiring urgent specialist appointments. Eye+dot’s automated triage was found to have moderate-substantial agreement with each of the 2 expert reviewers but lower agreement with the optometrist triage (Table 3).

**Table 2.** Disposition outcomes for the 53 patients with gold standard high-acuity symptomatology based on their online questionnaire result with sensitivity and specificity for identifying urgent cases (N=53).

Disposition urgency	Optometry triage (N=53)	Online triage (N=53)	*P* value
Within 24 h, n (%)	41 (77)	40 (75)	
Sensitivity, %	77	75	.99
Specificity, %	54	83	<.001
Within 48 h, n (%)	4 (8)	13 (25)	—^a^
Within a wk/routine/advice, n (%)	8 (15)	0 (0)	—

a Not applicable.

**Table 3.** Interrater reliability for urgent or nonurgent triage dispositions.

Pair	Cohen κ (95% CI)
Optometrist and eye+dot	0.26 (0.15-0.37)
Reviewer 1 and eye+dot	0.75 (0.64-0.86)
Reviewer 2 and eye+dot	0.61 (0.50-0.72)
Reviewer 1 and Reviewer 2	0.40 (0.28-0.52)

### Usability

The mean time taken to complete the online questionnaire was 4 minutes 57 seconds (SD 2 minutes 2 seconds; range 1 minute 30 seconds to 16 minutes 15 seconds). Overall, 147 out of 209 (70%) patients provided voluntary feedback about the eye+dot app via a 5-point visual Likert scale. Of these, 121 (82%) patients rated the app 4 or 5 (mean 4.27, SD 0.76) with 5 being the most positive rating. Of patients whose method of undertaking the questionnaire had been recorded, 71 of the 82 (87%) online respondents rated the app 4 or 5 compared to 9 of the 14 (64%) patients undertaking the questionnaire over the phone (mean 0.71, SD 4.39 and mean 4.21, SD 0.94 for online and telephone groups, respectively; *P*=.70 using the Mann-Whitney *U* test).

### Postimplementation Feedback

Qualitative postimplementation feedback responses to a semistructured email questionnaire by participating practices are shown in Textbox 1.

Textbox 1. **Semistructured postimplementation feedback from the 7 participating practices.**

**Were there any advantages of using the eye+dot tool?**

- The compiled symptom report improved documentation and supported onward referral
- The report provided useful documentation for records
- Helped reduce the need to interrupt optometrists undertaking scheduled work (3 Specsavers practices mentioned this)

**Were there any disadvantages of using the eye+dot tool?**

- Older patients were more hesitant to accept online triage
- Optometrists found it quite difficult to interpret the symptom report
- Patients often volunteered different symptomatology when telephoned
- Was not found helpful because there was already capacity to triage and book patient appointments without delay (at 2 independent practices)
- Increased triage time (at 2 independent practices)

**What were the barriers and enablers to implementing the innovation?**

- Uptake was affected by staffing shortages, changes, and the administrative burden of completing the study documentation
- Uptake was improved by the workflow being accepted as normal practice, with all eligible and able patients being routinely requested to complete the online questionnaire

**What improvements could be made to the eye+dot tool?**

- Recording eye laterality to enable comparison with previous optometric records
- A free text box for patients after the forced choice questions for clarifications, if needed

**Would your practice consider using an online triage tool in the future?**

-  Yes: 5 (4 Specsavers branches, one independent practice)
-  No: 2 (independent practices)

## Discussion

### Utility and Role of the Eye+Dot Triage Tool

Triage systems should ideally have both high sensitivity and high specificity for urgent cases to ensure timely access to care and efficient use of resources. This real-world study suggests that the eye+dot web app has similar sensitivity as community optometrists at identifying high-risk eye or vision symptomatology requiring specialist assessment within 24 hours and possibly superior specificity. The 3 patients whose symptoms were potentially sight- or life-threatening (based on the RCOphth guidelines; Textbox 1) were accurately identified by both optometric and eye+dot triage. Overall, 12 and 11 patients were underrated by optometry triage and eye.dot triage, respectively, compared to the gold-standard hospital clinician disposition based on review of the eye+dot report. In all 11 cases underrated by eye+dot, the disposition was for specialist review within 48 hours. Of the 12 patients who underrated the optometrists, 4 were given appointments within 48 hours and the remainder routine appointments.

There was substantial correlation between the urgency suggested by eye+dot and expert hospital clinician dispositions (κ=0.61 and 0.75). In contrast, there was only a slight correlation between the hospital clinicians and community optometrists (κ=0.26). Interestingly, most automated triage tools are found to have lower specificity than clinician triage for high-risk presentations, which does not align with our findings for the eye+dot triage tool relative to community optometrist triage [8,12,21]. In this study, the higher acuity triage decisions made by community optometrists may have been influenced by other factors, including additional information from the patient such as their level of anxiety and real-time knowledge of appointment availability. This suggests that human contact and decision-making still have an important role, even when an automatic triage tool is used.

Advantages of the real-world nature of our study design include the inclusion of individuals who might not usually contribute to such studies, understanding how technology can be implemented within service workflows, faster timelines, and cost efficiency. This may be particularly helpful for developers of innovative technology to rapidly understand its feasibility and usability in practice, identify improvements, and optimize its implementation model [22]. The qualitative post-implementation feedback has led to the inclusion of a question about symptom laterality and a free-text box for additional information after the forced-choice questions (Multimedia Appendix 2). A randomized controlled trial would have reduced the risk of bias and ensured optimal data quality, but would not have been feasible in busy, commercial optometry practices.

To the extent that these results represent a real improvement in eye+dot’s ability to accurately identify low-urgency cases relative to frontline human triage, they suggest that significant improvements in healthcare system efficiency could be achieved by using eye+dot or a similar tool to reduce overtriage of patients and consequent pressure on acute hospital services. However, the limitations of the pragmatic design of this study and the possible impact of population or region-specific factors mean that more work is required before definite or generalizable conclusions can be drawn from this observation. Of note, only 4% of patients in this study were advised to attend or contact the ED or EEC by the optometrists in this study, all of whom had received enhanced training as MECS providers. The proportion directed to hospital emergency services is likely to be higher among practices without MECS-trained clinicians.

While it is possible for a member of staff to take a patient through the questionnaire over the phone, only a minority of patients—14% of those for whom the delivery method was documented—chose to use this option. This form of delivery may be less feasible, given the time required. Regular smartphone use is less likely among older individuals, and such patients may prefer to have a 2-way conversation with a clinician if they are anxious about new symptoms [23,24]. These barriers to uptake make it important to ensure other means of triage are available.

The voluntary visual Likert score was not completed by 62 out of 209 or 30% of patients, but 82% (121/147) of those who did gave the eye+dot app a rating in the top 2 of 5 options, suggesting a reasonable level of acceptability for patients. The duration of the test was highly variable, with longer tests reflecting the complexity of the individuals’ symptoms and history. The use of web browser translation may have improved the triage experience and quality for individuals whose first language was not English, but this was not assessed during this study.

### Limitations

The uptake of digital triage technology in this study was influenced by burdensome study administrative requirements and staff turnover in optometry practices. Those recruiting the most patients had embedded the triage tool into their workflow, routinely inviting suitable patients to complete the questionnaire. Despite only having access to the symptom report (and not the automated triage disposition), all Specsavers practices and one independent optometry practice were interested in continuing to use the tool. Reported benefits included ease of documentation, avoiding interruptions to scheduled appointments, and more efficient MECS booking. The tool may be less useful in practices with more generous optometrist scheduling and greater capacity to triage effectively. Conversely, practices where staff adopt a cautious approach to triage due to time or resource constraints are likely to benefit the most from a triage tool with high specificity for urgent cases [5].

It is possible that patient selection was biased towards the inclusion of younger patients (mean age of 53.5, SD 17.5 y), although we do not have data on the average age of all patients presenting to these optometry practices across the relevant time period. Preferential inclusion of younger patients could potentially bias the range of eye symptoms and previous ophthalmic history elicited.

Retrospective review by expert clinicians is a common method used to establish the reference gold standard triage disposition in studies validating both paper and digital triage tools [9,25-27]. However, minor differences in the precise methodology can introduce varying limitations. In this study, the gold standard disposition may not be ideal for evaluation of optometrist triage as it relied purely on specialist review of symptom reports without an option for them to talk directly with the patient and without the eventual clinical diagnosis being known. On the other hand, the lack of clinical outcome data prevented outcome bias which might arise from knowing that a patient with high acuity red-flag symptoms eventually received a relatively benign diagnosis.

Finally, we were not able to estimate theoretical patient compliance with eye+dot’s automated triage advice from this study, as all patients were directed to services in line with their optometrist’s triage decision. Other studies have noted that patients advised to seek community health care by remote triage often present to the ED regardless [14]. However, since the eye+dot app is designed to support triage decisions made and communicated by clinical teams rather than issuing advice directly to the patient automatically, this risk may be relatively small.

### Conclusion

This real-world evaluation demonstrates that use of the eye+dot symptom-based online triage tool is feasible, acceptable to patients and staff, and can provide safe triage support, potentially improving triage efficiency and decreasing unnecessary hospital attendance if implemented in nonspecialist services.

These benefits may be even more pronounced if implemented in primary care settings without specialist triage resources or expertise. We are also currently evaluating the utility of the triage tool in hospital emergency services, both in supporting nurse-led triage for patients contacting the EEC service by phone and as a kiosk-based service for walk-in patients.

## Supplementary material

10.2196/77278 Multimedia Appendix 1 Symptom acuity table adapted from the Royal College of Ophthalmologists Emergency Eye Care Guidelines.

10.2196/77278 Multimedia Appendix 2 Example of an eye+dot symptom report and triage disposition.

10.2196/77278 Multimedia Appendix 3 Online preimplementation survey sent to optometrists registered with the Cambridgeshire Local Optometric Committee.

10.2196/77278 Multimedia Appendix 4 Royal College of Ophthalmologists (RCOphth) list of symptoms requiring emergency eyecare [16].

10.2196/77278 Multimedia Appendix 5 Visual Likert scale following completion of eye+dot questionnaire. The pictorial values were converted to numerical ones (least favorable=1, most favorable=5) for analysis.

10.2196/77278 Multimedia Appendix 6 Optometric triage outcomes and online triage tool–suggested dispositions. (A) Optometric triage outcomes. (B) Online triage tool suggested dispositions. Redder colors correspond to more urgent triage dispositions. Note that the eye+dot tool directs all urgent (within 24 h) dispositions to a national health service (NHS) hospital or minor eye condition services (MECS) services as urgent appointments are often not available privately or through the general ophthalmic service (GOS). N=209. ED: emergency department; EEC: emergency eye clinic; GOS: general ophthalmic service; MECS: minor eye condition service.

## Abbreviations

CUES: Community Urgent Eyecare Services
ED: emergency department
EEC: emergency eye clinic
GDPR: General Data Protection Regulation
MECS: Minor Eye Condition Services
NHS: National Health Service
RCOphth: Royal College of Ophthalmologists
