# Supplementary material for: Real-World Evaluation of the Eye+Dot Online Triage Support Tool in Community Optometry Practices: Mixed Methods Evaluation Study
Source: JMIR Hum Factors. 2026 Mar 16;13:e77278. doi: 10.2196/77278 (PMC12991188; doi:10.2196/77278)
Supplement: Multimedia Appendix 1 [file humanfactors-v13-e77278-s001.pdf]

Presenting problem	ED or EEC same day<br>High acuity -emergency	EEC or MECS within 24 hours<br>High acuity -urgent	Hospital or MECS 48-72 hours	Community service within the week
Trauma	Chemical injury<br>High velocity injury<br>Severe blunt trauma<br>Lid laceration	As A but >24 hours previously	Low velocity injury (e.g. fingernail or wind-blown foreign body	Other low risk injury
Red or painful eye	Severe pain ≥3 out of 5 with systemic symptoms<br>Generalised lid swelling with systemic symptoms	Severe pain ≥3 out of 5 without systemic symptoms<br>Pain ≥2 out of 5 in a contact lens wearer<br>Increasing pain within 2 weeks of an intra-ocular procedure<br>Photophobia to room lighting<br>Haloes in vision<br>Red eye with impaired vision (unable to read large print)	Pain rated ≤2 in CL wearer<br>Red eye with near normal vision (able to read normal print)	Gritty / itchy pink or red eye(s) with no reduction in vision (no CL history)<br>Eyelid cysts or localised lid swelling (able to open eye fully
Painless visual disturbance	Acute onset floaters (too many to count), with reduced vision (unable to read large print) or visual field defect<br><br>Acute onset diplopia, systemic symptoms	Acute onset of >10 floaters with risk factors (high myopia, VR history)<br>Painless reduction of vision within 2 weeks of an intra-ocular procedure<br>Acute onset double vision no systemic symptoms	Floaters low risk (<10, no sudden onset, no visual deterioration, no ophthalmic history)<br>Photopsia in isolation<br>Blurred vision low risk able to read titles of print, gradual onset<br>Sub-acute onset binocular diplopia	Gradual or>1 week onset of <10 floaters without visual deterioration
Painless visual deterioration	Acute deterioration in vision (counting fingers or worse) / field loss within 24 hours<br>Deterioration in vision (unable to read large print) associated with systemic symptoms, haloes or photophobia to normal room lighting	Acute reduction in vision >24 unable to read	Sub-acute visual deterioration< 1 week, not able to read print	Gradual reduction in vision for a week unable to read normal print
External eye	Unable to open lids, systemically unwell	Swelling of whole lid with erythema, limited lid opening	Swelling of whole lid with erythema	Localised swelling
