# Supplementary material for: Real-World Evaluation of the Eye+Dot Online Triage Support Tool in Community Optometry Practices: Mixed Methods Evaluation Study
Source: JMIR Hum Factors. 2026 Mar 16;13:e77278. doi: 10.2196/77278 (PMC12991188; doi:10.2196/77278)
Supplement: Multimedia Appendix 2 [file humanfactors-v13-e77278-s002.pdf]

Digital triage tool survey

We would like to have your views about a new online triage tool for use in individuals with a recent onset of new eye symptoms. A link to the online triage tool can be sent from the practice to the individual by SMS or email. This leads them to a branching logic questionnaire of up to 25 individualised questions. The tool compiles a downloadable symptom and ophthalmic history report which is automatically emailed to the requestor on completion. The tool can also provide a suggested triage disposition in terms of provider and urgency.
This survey will take approximately 5 minutes, many thanks for your time.

* Required

1. What is the name (and site, if relevant) of your practice? *

Enter your answer

2. What is your role? *

- ◯ Receptionist
- ◯ Dispensing optician
- ◯ Optometrist
- ◯ Administrator / manager
- ◯ Optical assistant
- ◯ Other

3. What is your age -choose from the following ranges *

- Under 20
- 21-29
- 30-39
- 40-49
- 50-59
- 60 and over

4. Approximately how many individuals with new (onset within the last seven days) eye symptoms do you speak to and/or triage per week? *

The value must be a number

5. Put the following in the order of the way in which your practice is contacted for urgent eye problems, with the most likely at the top *

the individual has been asked to contact the practice by their GP practice

the individual has been asked to contact the practice by NHS111

the individual has been asked to contact the practice by secondary care teams

the individual has walked-in for advice without previously being asked to do so

the individual has emailed or phoned without previously being asked to do so

6. Put the following in order of the frequency of outcomes after such contacts, with the most likely at the top *

advice to attend Emergency Department / Eye Emergency without optometric examination

advice to see a pharmacist or purchase drops without optometric examination

advice to contact GP without optometric examination

advice to contact NHS111 without optometric examination

advice to make MECS optometry appointment

advice to make GOS or private optometry appointment

7. Does your practice currently use a paper-based triage tool? *

- ◯ Yes
- ◯ No

8. Please indicate your opinion about the following statements with reference to individuals contacting your practice with recent onset eye symptoms: *

	Strongly Disgree	Disagree	Neutral	Agree	Strongly Agre
I find triage difficult	◯	◯	◯	◯	◯
I don't have the time to triage effectively	◯	◯	◯	◯	◯
I find it difficult giving advice without an examination	◯	◯	◯	◯	◯
I would value a triage support tool	◯	◯	◯	◯	◯
I am prepared to learn how to use an online triage tool	◯	◯	◯	◯	◯

	Strongly Disgree	Disagree	Neutral	Agree	Strongly Agree
Individuals with urgent eye problems will be willing and able to use an online triage tool	◯	◯	◯	◯	◯
A triage tool will improve patient care	◯	◯	◯	◯	◯
An electronic symptom and ophthalmic history report will make onward referral easier	◯	◯	◯	◯	◯

9. Would like to be contacted with more information about how to participate in the implementation of eye.dot? *

◯ Yes

◯ No

10. If so, please enter your name and email address here

Enter your answer

Never give out your password. Report abuse
