# Supplementary material for: Real-World Evaluation of the Eye+Dot Online Triage Support Tool in Community Optometry Practices: Mixed Methods Evaluation Study
Source: JMIR Hum Factors. 2026 Mar 16;13:e77278. doi: 10.2196/77278 (PMC12991188; doi:10.2196/77278)
Supplement: Multimedia Appendix 3 [file humanfactors-v13-e77278-s003.pdf]

eye+dot®
digital online triage

results

Order Number: XX250418/001
Completed: 18th April 2025 18:33
Duration: 3 min 08 secs

Age: 45

1.	**What best describes your eye problem?**<br>Red or painful eye (with or without visual disturbance)
2.	**The problem is affecting:**<br>The right eye
3.	**Is the affected eye bloodshot or red?**<br>The eye is bloodshot
4.	**How much of the eye is affected?**<br>The whole eye
5.	**How long has the eye been bloodshot or red?**<br>Less than 24 hours
6.	**Are the eyelids of the affected eye swollen or puffy?**<br>The eyelids are not swollen or puffy
7.	**Are you getting any watering or discharge from your eye?**<br>Tears and watering only
8.	**Is the problem causing pain or discomfort?**<br>There is a constant dull ache
9.	**How severe is the pain from 0-5 (no pain is 0 and excruciating pain is 5)?**<br>The pain is severe: 4 or 5 out of 5
10.	**Is the level of discomfort or pain changing?**<br>The discomfort or pain is getting worse
11.	**Have you become more sensitive to bright light?**<br>I am more sensitive to light than usual
12.	**How severe is your sensitivity to light?**<br>It is painful to look at normal room lights
13.	**Is your vision (with glasses correction if worn) worse than usual?**<br>No, I don't think that my vision has been affected
14.	**Are you seeing haloes around lights?**<br>I have not noticed haloes around bright lights
15.	**How are you feeling in yourself?**<br>Apart from my eye problem, I otherwise feel normal
16.	**Do you regularly wear contact lenses?**<br>I don't wear contact lenses
17.	**Have you had a recent hospital eye examination or procedure on the affected eye(s)?**<br>No
18.	**Have you had an eye condition before?**<br>Yes, I have had a problem previously
19.	**What conditions have you had before? (select all that apply)**<br>Eye inflammation: iritis, uveitis, scleritis in either eye
20.	**Are you currently using any medication (including non-prescription medication) for your eyes?**<br>I am not using any medication for my eyes
21.	**Do you suffer from any of the following? (select all that apply)**<br>*Choices: allergies/herpes/RA/autoimmune/connective tissue/thyroid/facial weakness/sinusitis*<br>None of the above

Additional Information Provided:

Feels like my previous attack of iritis

Suggested disposition:

Suitable to be seen in Emergency Department or Emergency Eye Clinic the same day.
