# Supplementary figures and images for: Real-World Evaluation of the Eye+Dot Online Triage Support Tool in Community Optometry Practices: Mixed Methods Evaluation Study
Source: JMIR Hum Factors. 2026 Mar 16;13:e77278. doi: 10.2196/77278 (PMC12991188; doi:10.2196/77278)

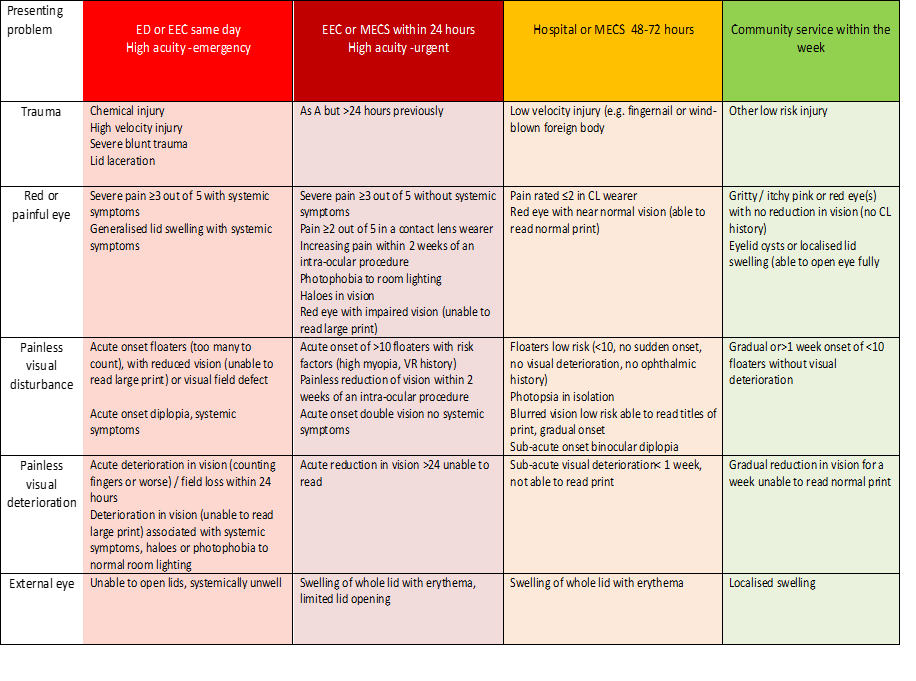

Supplement: Multimedia Appendix 4 [file humanfactors-v13-e77278-s004.png]

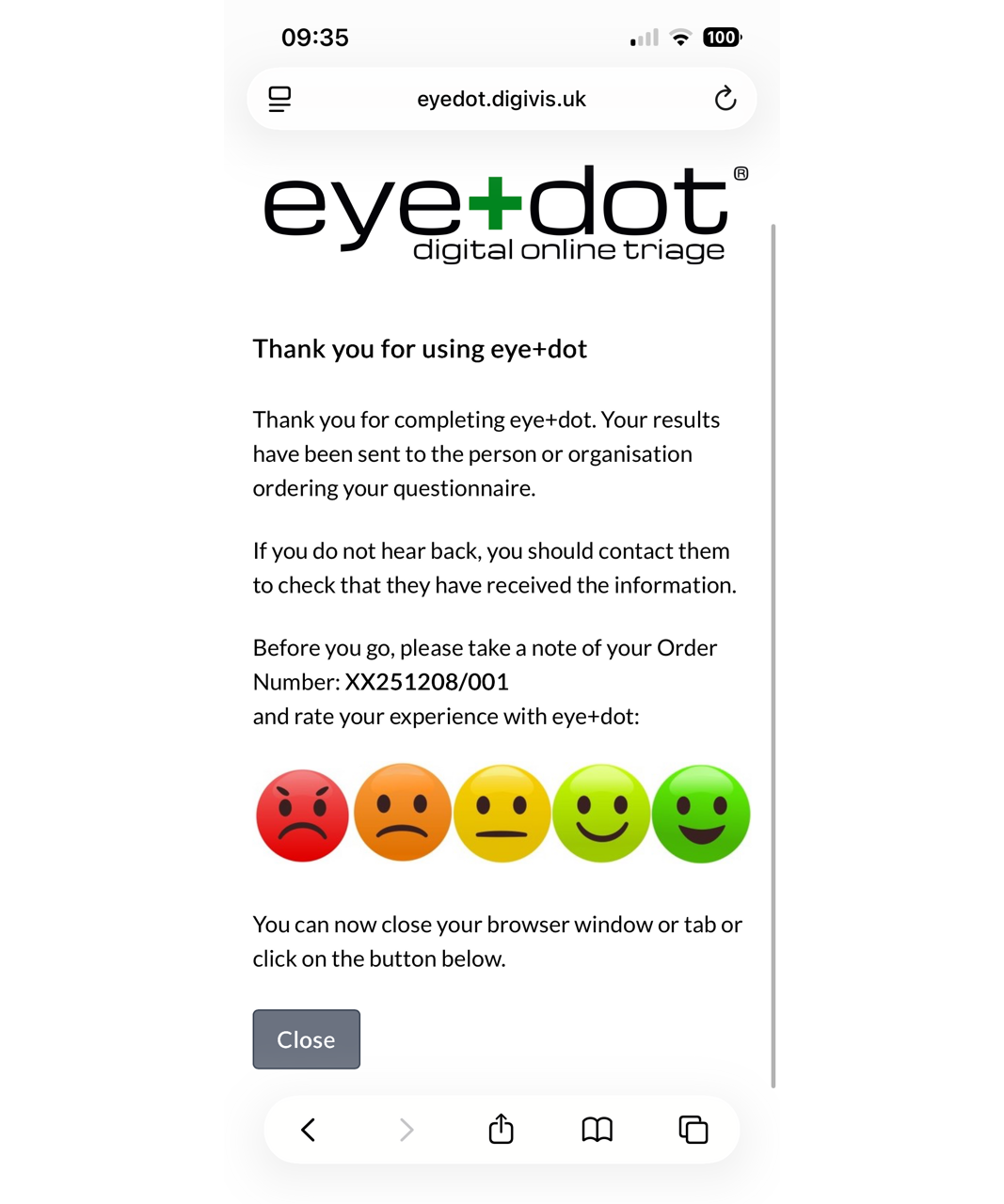

Supplement: Multimedia Appendix 5 [file humanfactors-v13-e77278-s005.PNG]

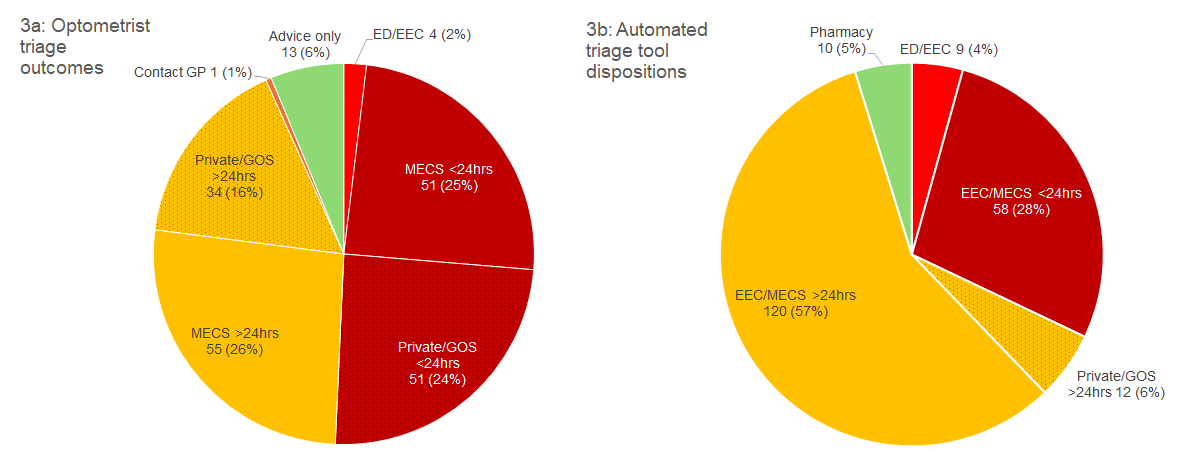

Supplement: Multimedia Appendix 6 [file humanfactors-v13-e77278-s006.png]
